# A Cause of Anæmia

**Published:** 1879-06

**Authors:** 


### A CAUSE OF ANÆMIA.

As soon as the change is made in the dress, from that of a child, custom demands also that she should be protected by veil and gloves from the rays of the sun, and she soon becomes as blanched as a well cultivated celery stalk. And since the blood needs the chemical effect of sun light acting directly on the skin, anæmia is established chiefly from the deprivation. This state of the blood is a potent factor in the generation of all diseases depending on impaired nutrition, and entails conditions likely to baffle all medical effort at their removal during menstrual life of the female.—*Emmet's Gynæcology.*

